# Predictors of Instrumental Activities of Daily Living Performance in Patients with Stroke

**DOI:** 10.1155/2021/6675680

**Published:** 2021-02-27

**Authors:** Amin Ghaffari, Hamid Reza Rostami, Malahat Akbarfahimi

**Affiliations:** ^1^Department of Occupational Therapy, School of Rehabilitation Sciences, Iran University of Medical Sciences, Tehran, Iran; ^2^Department of Occupational Therapy, Musculoskeletal Research Center, School of Rehabilitation Sciences, Isfahan University of Medical Sciences, Isfahan, Iran

## Abstract

**Objective:**

Instrumental activities of daily livings are important for independent living and active participation in the community. The present study is aimed at determining factors predicting instrumental activities of daily living performance in patients with stroke.

**Methods:**

In this cross-sectional study, a convenient sample of 90 patients with stroke entered from five occupational therapy centers, which were selected based on the cluster randomization method. Lawton IADL scale, Barthel Index, Trail Making Test (A and B), Digit span subtest of Wechsler memory scale, Motorcity index, and Beck Depression Inventory-II were used to investigate the study's aim. Statistical analyses were performed using independent sample *t*-test, one-way ANOVA, Pearson correlation, and multiple linear regression analysis.

**Results:**

Age (*r* = −0.384, *p* < 0.001), memory (*r* = 0.565, *p* < 0.001), basic activities of daily living (*r* = 0.818, *p* < 0.001), depression (*r* = −0.758, *p* < 0.001), Trial Making Test _(B-A)_ (*r* = −0.614, *p* < 0.001), and motoricity index (*r* = 0.670, *p* < 0.001) were significantly associated with instrumental activities of daily living performance.

**Conclusions:**

Basic activities of daily living were the strongest predictor of IADL's performance. Age, TMT _(B-A)_, and depression were orderly the next strongest predictors. Stroke patients with more dependency in basic activities of daily living, older age, cognitive impairment, and depression are more opted to be dependent in instrumental activities of daily living and as a result, less participation in home and community affairs.

## 1. Introduction

Stroke is the most common and debilitating neurological disorder among adults [1]. According to the Global Burden of Disease, Injury, and Risk Factor, stroke is the third leading cause of disability in the world [2]. Occupations are various kinds of meaningful and purposeful activities in which individuals engage, reflect, and support persons' interests and skills [3]. Areas of occupations consist of basic activities of daily living (BADLs), instrumental activities of daily living (IADLs), sleep, education, work, play, leisure, and social participation [4]. BADLs and IADLs are examples of domains limited following a stroke which could result in restriction of participation in different life situations [5]. BADLs and IADLs encompass a wide range of activities important for living independently in the home and community [6]. IADLs are more complicated than BADLs and require more complex interactions within the environment [7], which makes IADLs more valued in assessing levels of well-being in patients with stroke than BADLs [8, 10]. Disabilities and limitations in performing IADLs independently can serve as an early detector of losing BADL function and independent living [11]; so, IADL limitations can negatively influence participation, well-being, and quality of life in patients with stroke [12].

Previous studies just considered predictors of BADLs following stroke. For example, Veerbeek et al. found baseline neurological status, upper limb paresis, and age as early predictors of BADL abilities after stroke [13]. Among different basic activities of daily living, Gialanella et al. mentioned social interaction, grooming, upper body dressing, and bowel control as the most important predictors for functional outcomes following stroke [14]. Despite the importance of IADLs for independent living and active participation in the community, there is a lack of literature on IADL predictors poststroke. Babulal et al. determined cognitive and mood impairments at two weeks poststroke as important predictors for IADL's performance during the first three months after stroke [15]. However, different factors influence IADL's performance including cognitive abilities (e.g., attention and executive functions), motor abilities (e.g., balance and dexterity), psychological abilities (e.g., motivation and depression) [8, 9], and contextual factors (personal factors including age, gender, and education and environmental factors comprising social, cultural, and economic) [16], which were neglected in the Babulal's study. Based on the literature, there is a significant relationship between IADL's performance and factors such as depression [17], cognition [18], age, and gender [19] in patients with stroke.

Considering the mentioned issues, maximizing the abilities and capabilities of patients with stroke in performing IADLs as independently as possible could be the main goal for occupational therapy and rehabilitation interventions. Stroke patients with problem-solving skills, social skills, and complex environmental interactions and those motivated enough to participate in the community, IADL performance is necessary for independent performance [20, 21]. For succeeding in this goal, determining predictors for performing IADLs could be a basic requirement. Based on the present literature, further studies can help to clarify predictors of IADL performance poststroke to make the person with stroke as an active participant in the community as soon as possible. Therefore, we aimed to determine predictors of IADLs performance in patients with stroke, among cognitive, motor, and psychological features in addition to contextual factors such as personal and environmental factors.

## 2. Methods

In this cross-sectional study, a convenient sample of 90 people with stroke was recruited from March 2019 to December 2019. Inclusion criteria consisted of first-ever stroke diagnosed by a neurologist, right hand dominance, age between 30 and 80 years old, successful formal education ≥ 9 years, and normal or corrected-to-normal visual acuity. Patients with a history of transient ischemic attack, other acute or chronic neurological and psychiatric disorders, unilateral neglect, aphasia, and any history of depression or taking antidepressants prior to stroke were excluded. The exclusion and inclusion criteria were controlled by neurologists and medical records.

To consider the geographical distribution, participants were entered into the study from five occupational therapy clinics distributed in different regions of aimed research's city (Tehran) by the principal investigator. Occupational therapy centers were selected based on the cluster randomization method. The study protocol was approved by the local ethics committee (IR.IUMS.REC1395.9411355005).

In the first step after signing informed consent by eligible people with stroke, participants' demographic and clinical information including age, gender, time since stroke, educational level, marital status, job, affected limb, cigarette smoking habits, diabetes mellitus, cardiac diseases, and blood pressure were gathered by the principal investigator. Then, an occupational therapist blinded to the study's aims measured the psychological, cognitive, motor, and activities of daily living domains using different assessment tools, which were administered in a random order.

Lawton instrumental activities of daily living scale were used to measure performance in IADLs such as using the telephone, shopping, food preparation, housekeeping, laundry, transportation, responsibility for own medication, and ability to handle finances. Its scores range from 0 (low function, dependent) to 8 (high function, independent) [7].

Cognitive domain was measured using the Trail Making Test (TMT) and Wechsler memory scale (WMS-R). TMT assesses executive functions such as mental flexibility, visual attention, and task switching with a motor component in two parts including TMT-A and TMT-B [22]. Participants match the numbered circles orderly as fast as possible in TMT-A and alternatively match the circled numbers and letters in TMT-B. The time required to complete each task is recorded in seconds. The difference in score between TMT-A and TMT-B (B-A) was calculated as indices of executive functions [23]. Scores for TMT (B-A) range from less than 58 (equals to 4) to bigger than 186 (equals to 0). Also, the digit span subtest of WMS-R was used to measure verbal memory in the form of forward and backward numbers with scores ranging from 3 to 8 for forward and 3 to 7 for backward numbers. The total score of the digit span subtest is the sum of both parts [24]. The total score of WMS-R ranges from 6 to 15.

Motoricity index was used to evaluate arm motor ability (pinch grip, elbow flexion, shoulder abduction), leg motor ability (ankle dorsiflexion, knee extension, hip flexion), and trunk control (rolling to the weak side, rolling to the strong side, sitting up from lying down, balance in sitting position). Its total score ranges from 0 to 200 [25].

Barthel Index measured the performance of participants in basic activities of daily living such as bowel and bladder function, grooming, toilet use, feeding, transfer, mobility, dressing, steps, and bathing. Its scores range from 0 to 100 which means complete dependency to complete independence [26].

Beck Depression Inventory-II (BDI-II) was used to assess the severity of depression, which consists of 21 items concerning people's feelings in different situations during the last week. Each item scores from 0 to 3. The cut-off point for depression in the Iranian population is 15 [27]. Its total score ranges from 0 to 63.

Data were analyzed using SPSS® Version 16 (SPSS Inc., Chicago, IL, USA). The chi-square test and Fisher exact test were used to compare categorical variables between groups and independent *t*-test for continuous variables. Pearson correlation coefficient was used to analyze the relationship between IADL performance and other demographic, clinical, cognitive, motor, and psychological features in addition to the contextual factors. Finally, multiple linear regression analysis was performed to identify the variables associated with IADL performance. Significant variables in independent sample *t*-test, one-way ANOVA, and Pearson correlation were subjected to multiple linear regression analysis, and backward elimination was performed. The statistically significant level was set at 0.05. Adjusted *R*^2^ was used to determine the variance of IADL performance.

## 3. Results

Ninety participants acquired the necessary criteria for entering into the study; however, the analysis was performed on 82 (96.6%) participants due to incomplete and incorrect questionnaires by eight participants who were excluded from data analysis. Descriptive information is mentioned in Table 1. The mean and standard deviation of IADL's performance was 4.00 ± 2.22 (Table 2).

Correlation analysis by Pearson correlation coefficient revealed age (*r* = −0.384, *p* < 0.001), memory (*r* = 0.565, *p* < 0.001), BADLs performance (*r* = 0.818, *p* < 0.001), depression (*r* = −0.758, *p* < 0.001), TMT _(B-A)_ (*r* = −0.614, *p* < 0.001), and motor function (*r* = 0.670, *p* < 0.001) significantly associated with IADL performance (Table 3). Independent sample *t*-test and one-way ANOVA showed significant differences in IADL performance among participants with different levels of demographic and clinical characteristics (educational level (*p* = 0.001), using assistive device (*p* = 0.024), job status (*p* = 0.019), and smoking (*p* = 0.002)) (Table 3).

Age, cognitive statue (Wechsler memory scale and TMT _(B-A)_), BADL performance, depression, and motoricity index were independent variables for linear regression analysis. Finally, age (*t* = −3.303, *p* < 0.05), TMT _(B-A)_ (*t* = −2.223, *p* < 0.05), BADL performance (*t* = 3.152, *p* < 0.05), and depression (*t* = −2.036, *p* < 0.05) were revealed as significant predictors for IADL performance. The multiple correlation coefficient (*R*) was 0.861, and the adjusted *R*^2^ was 74.13% (*F* = 35.759, *p* < 0.001) (Table 4).

Stepwise analysis of the linear regression model with significant variables showed BADL's performance as the strongest predictor of IADL's performance. Age, TMT _(B-A)_, and depression were orderly the next strongest predictors (Table 5).

## 4. Discussion

We investigated factors influencing IADL's performance in patients with stroke. Based on our findings, age, TMT _(B-A)_, BADL, and depression were predictors of IADL while BADLs performance emerged as the most important factor predicting IADL performance. Both BADLs and IADLs are crucial for independent living. However, BADLs require more basic sensory motor, cognitive, and psychosocial abilities which make them prerequisites for succeeding in IADLs which are more advanced and complicated abilities needed for independent home and community living and participation [28]. Although stroke patients may acquire different levels of IADLs and BADL performance, but patients generally gain gradual independence in BADLs first [17]. Katz et al. found that more complex activities such as IADLs would be lost first following neurologic disorders, while more basic activities such as BADLs could be saved until the last stages of most diseases [29]. Therefore, paying special attention to improving BADLs as a prerequisite for the future independence of patients in home and community participation should be considered in the rehabilitation process.

The next predictor for IADL's performance in our study was age, which showed a negative correlation with IADL performance; in other words, older patients with stroke will experience more limitations in IADL performance. In a recent study, Ćwirlej-Sozańska et al. found with each subsequent year of life, the odds of having problems with BADLs increased by 8%, and the odds of having problems with IADLs increased by 10% [30]. In addition, Connolly et al. reported an approximately two and one-half folds to increase in the 75–79 years age group and a four folds increase in the 80 and older age group compared to the 65–69 years age group for the risk of BADLs and IADL performance difficulties [31]. Older patients with stroke experience a different set of stroke risk factors because of negative changes in body functions and structures; thus, they are more opted to suffer from complications poststroke compared to younger patients [32].

Also, our results showed that the Wechsler memory scale and TMT (B-A) were significantly associated with IADL's performance; but, only TMT (B-A) was a predictor of independence in IADL performance. This finding is consistent with the results of the Babulal et al. that using the TMT test found cognitive impairments predict 48% of the variance in IADL performance [15]. One of the areas assessed by TMT test is executive functions. Since different domains of IADL performance such as organization, sequencing, judgment, and completion need a lot of executive functions processes, IADL performance and executive functions are strongly associated [33]. Despite significant relationship, verbal section of Wechsler memory scale could not predict IADL performance in our study may be because of a variety of activities which some of them needed verbal memory (using the telephone, shopping, transportation, and the ability to handle finances) and others did not require verbal memory (food preparation, housekeeping, laundry, and responsibility for own medication).

Different cognitive abilities could be affected following stroke (incidence of deficits in episodic memory about 55%, deficits in executive functions up to 40%, language deficits about 23%) [34]. Deficits in episodic memory [35], executive functions [36], visual attention [37], and language [38] are associated with difficulties in performing BADLs and IADLs. Zinn et al. found weaker IADL recovery and performance in cognitively impaired patients with stroke [39]. In addition, according to the literature, IADL performance is sensitive to early cognitive decline whereas body physical ability is often a significant predictor for BADL ability [40, 41]. Therefore, paying special attention to all domains of cognitive rehabilitation seems crucial for maximizing IADL's performance and home and community participation in patients with stroke.

Another predictive factor for IADL's performance in our study was depression; in other words, patients with more severe depression were less independent in IADL's performance. Lai et al. found stroke patients with depressive symptoms achieved independence in BADLs and IADLs slower compared to patients without depression [17]. Basic activities of daily living and trunk motor control are reported as two discriminative factors for distinguishing patients with poststroke depression [42]. Furthermore, impaired activities such as shopping and preparing meals due to cognitive impairments have been reported as a prominent disorder in the presence of depressive symptoms [18]. Therefore, poststroke symptoms of depression and specific cognitive dysfunctions need to be recognized and appropriately treated for enhancing functional outcomes.

Despite the significant correlation between motor function and independence in IADL performance in our study in line with previous literature [43, 44], motor function was not a predictor of IADL performance in our study. Motoricity index which was used in the present study for measuring motor function is a questionnaire assessing basic motor functions that are more important for BADLs [45]. However as it was mentioned, IADLs are more sophisticated activities with different and more complicated movements. Li et al. mentioned that a functional motor test such as Action Research Arm Test can predict IADL's performance in patients with stroke [46]. The question arising here is if the motor function is not a predictor of IADL performance as it is reported in our study or using a functional assessment tool such as Action Research Arm Test could make a change in our results about prediction validity of motor function on IADL's performance. This issue needs to be investigated in future studies.

Among the demographic and clinical information, smoking, using assistive devices, low educational level, and lack of job had a significant negative correlation with independence in IADL's performance, although no one of them was a predictor of IADL's performance in our study. In general, the results of our study confirm the findings of Lindström et al. [47] and Howard et al. [48] which found a significantly higher probability of return to participation and independence among persons with a higher socioeconomic class. Involving in a job needs more participation in the community per se. Since IADLs involve activities more correlated with independent living and community participation [49], the association between IADLs and involved in a job seems logical. Similar to involving in a job, higher educational level involves more complicated activities that are more correlated with community participation [50]. Previous literature has introduced education and income as independent predictors of returning to work poststroke [51]. A negative significant correlation between using assistive devices and independence in IADL's performance maybe because of the association of using assistive devices with decreased balance, strength, mobility, and stair-climbing power, in addition to slower gait speed and increased risk for falling [52, 53]. It is revealed in previous studies that using assistive devices is correlated with greater degrees of body functions and structures impairments and more functional dependency in activities of daily living [52, 54].

Some limitations in our study need to be considered in future studies. The cross-sectional design of the present study limited its ability to predict the causal relationship among variables. Entering patients with some of our exclusion criteria in future studies can make the results of the present study more generalizable. Due to mentioned limitations, our results should be considered cautiously.

## 5. Conclusion

Stroke patients with more dependency in basic activities of daily living, older age, cognitive impairment, and depression are more opted to be dependent in instrumental activities of daily living and as a result, less participation in home and community affairs. So, occupational therapists and other rehabilitation experts should screen at-risk people with stroke and implement interventions necessary for improving instrumental activities of daily living and community participation as soon as possible.

## Figures and Tables

**Table 1:** Descriptive information about the demographic and clinical characteristics of participants.

Variable	Number (%)	Variable	Number (%)	Variable	Number (%)
Gender		Education		Marital status	
Male	39 (47.6%)	Below diploma	31 (37.8%)	Single	12 (14.6%)
Female	43 (52.4%)	Diploma	29 (35.4%)	Married	70 (85.4%)
		Above diploma	22 (26.8%)		
Hemiplegia		Job		Assistive device	
Right	30 (36.6%)	House making	25 (30.5%)	Yes	46 (56.1%)
		Retired	38 (46.3%)		
Left	52 (63.4%)	Employed	19 (23.2%)	No	36 (43.9%)
Smoking		Diabetes		Cardiovascular disease	
Yes	19 (23.2%)	Yes	20 (24.4%)	Yes	27 (32.9%)
No	63 (76.8%)	No	62 (75.6%)	No	55 (67.1%)

**Table 2:** Mean and standard deviation of variables (*N* = 82).

Variables	Mean	SD
Motor function	147.00	35.20
TMT _(B-A)_	115.87	53.94
Age	55.96	13.47
Memory	8.36	2.18
BADL	67.92	26.63
Depression	19.79	13.30
IADL	4.00	2.22

**Table 3:** Univariate analysis and correlation of different variables with instrumental activities of daily living.

Variables	*t*/*F*	*r*	*p* value
Age		-0.384	0.000
Memory		0.565	0.000
BADL		0.818	0.000
Depression		-0.758	0.000
TMT _(B-A)_		-0.614	0.000
Motor function		0.670	0.000
Educational level	7.47		0.001
Gender	0.245		0.807
Marital status	-0.822		0.413
Job	4.150		0.019
Hemiplegic side	-0.439		0.663
Use assistive device	-2.309		0.024
Smoking	-3.208		0.002
Diabetes	0.374		0.709
Blood pressure	-0.051		0.960
Cardiovascular disease	-0.071		0.944

**Table 4:** Linear regression analysis of IADL level.

Variables	Beta	*t*	*p* value
Motor function	0.088	0.818	0.416
TMT _(B-A)_	-0.179	-2.223	0.029
Age	-0.207	-3.303	0.001
Memory	-0.079	-0.904	0.369
BADL	0.446	3.152	0.002
Depression	-0.228	-2.036	0.045

**Table 5:** Stepwise analysis of linear regression model for IADL.

Model	Beta	*T*	*p* value
BADL	0.818	12.73	0.000
BADL	0.770	12.28	0.000
Age	-0.199	-3.17	0.002
BADL	0.674	8.74	0.000
Age	-0.192	-3.11	0.003
TMT _(B-A)_	-0.159	-2.08	0.041
BADL	0.488	4.13	0.000
Age	-0.192	-3.18	0.002
TMT _(B-A)_	-0.153	-2.04	0.044
Depression	-0.223	-2.04	0.045

## Data Availability

All data used to support the results of this study are included in the article.
